# A Mutation in the Myostatin Gene Increases Muscle Mass and Enhances Racing Performance in Heterozygote Dogs

**DOI:** 10.1371/journal.pgen.0030079

**Published:** 2007-05-25

**Authors:** Dana S Mosher, Pascale Quignon, Carlos D Bustamante, Nathan B Sutter, Cathryn S Mellersh, Heidi G Parker, Elaine A Ostrander

**Affiliations:** 1 National Human Genome Research Institute, National Institutes of Health, Bethesda, Maryland, United States of America; 2 Department of Biological Statistics and Computational Biology, Cornell University, Ithaca, New York, United States of America; 3 Animal Health Trust, Center for Preventive Medicine, Newmarket, United Kingdom; Northwestern University, United States of America

## Abstract

Double muscling is a trait previously described in several mammalian species including cattle and sheep and is caused by mutations in the myostatin *(MSTN)* gene (previously referred to as *GDF8*). Here we describe a new mutation in *MSTN* found in the whippet dog breed that results in a double-muscled phenotype known as the “bully” whippet. Individuals with this phenotype carry two copies of a two-base-pair deletion in the third exon of *MSTN* leading to a premature stop codon at amino acid 313. Individuals carrying only one copy of the mutation are, on average, more muscular than wild-type individuals (*p* = 7.43 × 10^−6^; Kruskal-Wallis Test) and are significantly faster than individuals carrying the wild-type genotype in competitive racing events (Kendall's nonparametric measure, τ = 0.3619; *p* ≈ 0.00028). These results highlight the utility of performance-enhancing polymorphisms, marking the first time a mutation in *MSTN* has been quantitatively linked to increased athletic performance.

## Author Summary

An individual's genetic profile can play a role in defining their natural skills and talents. The canine species presents an excellent system in which to find such associative genes. The purebred dog has a long history of selective breeding, which has produced specific breeds of extraordinary strength, intelligence, and speed. We have discovered a mutation in the canine myostatin gene, a negative regulator of muscle mass, which affects muscle composition, and hence racing speed, in whippets. Dogs that possess a single copy of this mutation are more muscled than normal and are among the fastest dogs in competitive racing events. However, dogs with two copies of the same mutation are grossly overmuscled, superficially resembling double-muscled cattle known to possess similar mutations. This result is the first to quantitatively link a mutation in the myostatin gene to athletic performance. Further, it emphasizes what is sure to be a growing area of research for performance-enhancing polymorphisms in competitive athletics. Future implications include screening for myostatin mutations among elite athletes. However, as little is known about the health issues and potential risks associated with being a myostatin-mutation carrier, research in this arena should proceed with extreme caution.

## Introduction

**Figure 1:**
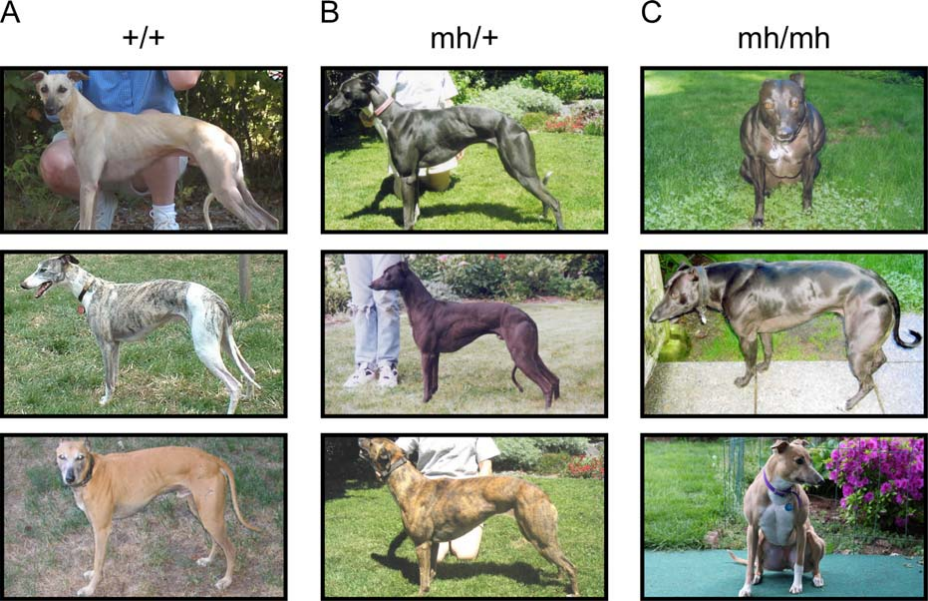
Comparison of Whippets with Each of the Three Potential Genotypes (A) Dogs have two copies of the wild-type allele (+/+). (B) Dogs are heterozygous with one wild-type allele and one mutant cys → stop allele (*mh/+)*. (C) Dogs are homozygous for the mutant allele with two copies of the cys → stop mutation *(mh/mh).* All photos represent unique individuals except for the top and middle panels in the righthand column.

The wide variety of behaviors and morphological types exhibited among dog breeds and the overall low genetic diversity within each breed make the dog an excellent genetic system for mapping traits of interest [1,2]. Recently, owners of whippets, an established racing-dog breed, have reported a phenotype of heavy muscling occurring within the breed (http://www.k9community.co.uk/forums/index.php). The typical whippet is similar in conformation to the greyhound, a medium-sized sighthound, weighing about 9 kg and characterized by a slim build, long neck, small head, and pointed snout (Figure 1A) [3]. Heavily muscled dogs, termed “bully” whippets by breeders, have broad chests and unusually well-developed leg and neck musculature (Figure 1C). “Bully” whippets are easily distinguished from their normal littermates based on physical appearance alone (compare Figure 1A and 1C). Owners report that “bully” whippets do not have any health abnormalities other than muscle cramping in the shoulder and thigh. However, the dogs are often euthanized at an early age as they do not conform to the American Kennel Club breed standard. In addition, about 50% of “bully” whippets have a distinctive overbite.

The “bully” whippet phenotype is reminiscent of the double muscling phenotype seen in other species that is caused by mutations in the myostatin *(MSTN)* gene. Such variants have been observed in mice [4], cattle [5,6], sheep [7], and human, the latter described once in a German boy [8].

The myostatin protein has been shown to affect both the amount and composition of muscle fibers. For instance, the muscle mass of *Mstn* knockout mice is two to three times greater than that of wild-type mice [9]. Furthermore, the sequence of the *MSTN* gene is relatively conserved across species [9]. Therefore, we chose to interrogate the *MSTN* gene for possible mutations resulting in the “bully” whippet phenotype.

## Results

### MSTN Genotypes in the Whippet

**Figure 2:**
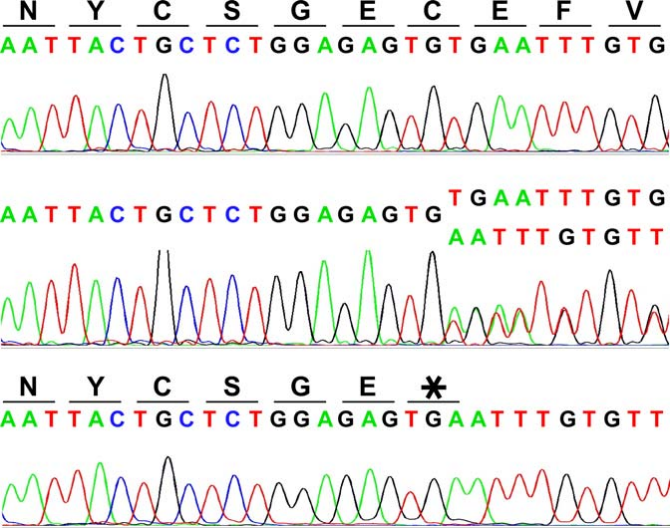
A 2-bp Deletion at Nucleotides 939 and 940 of the Canine *MSTN* Coding Sequence This deletion results in a cysteine→stop codon change at amino acid 313. The top panel shows the sequence trace of a wild-type (*+/+*) individual in the region of the mutation. The middle panel shows the sequence trace of a *mh/+* individual with a single copy of both the wild-type and mutant alleles. The bottom panel shows the sequence trace from a homozygote “bully” dog of the *mh/mh* genotype. The amino acid sequences for *+/+* and *mh/mh* individuals are located above each trace.

We sequenced the three exons and the majority of introns of the *MSTN* gene in an initial set of 22 whippets. A 2-bp deletion was discovered in the third exon of the *MSTN* gene (Figure 2). This deletion removes nucleotides 939 and 940 within exon three and leads to a premature stop codon at amino acid 313 instead of the normal cysteine, removing 63 aa from the predicted 375-aa protein. The lost cysteine is one of several highly conserved cysteines known to form disulfide dimers required for protein function [9].

Of the 22 whippets sequenced, all “bully” whippets tested (*n* = 4) were homozygous for the deletion *(mh/mh)* while all dogs that sired or whelped a “bully” whippet (*n* = 5) were heterozygous for the deletion (*mh/+*). None of the initial set of 13 normal-appearing whippets that lacked a family history of the “bully” phenotype carried the deletion; these dogs were designated wild type (*+/+*). An additional set of DNA samples from 146 whippets (both racers and nonracers) were collected at racing events and through the mail without regard to the dogs' family histories of the “bully” phenotype. These were sequenced across exon three to determine the frequency of the 2-bp mutation among the dogs sampled. Of these, two were homozygous for the deletion, 20 were heterozygous, and the remaining 124 did not carry the deletion.

### Mode of Inheritance and Heterozygote Phenotypes

**Figure 3:**
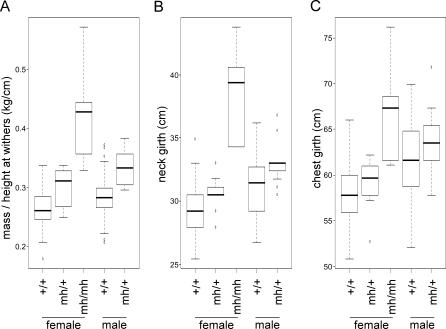
Variation in Musculature among Whippets of the Three Potential Genotypes Whippets homozygous for the cys → stop mutation have a higher mass-to-height ratio (A), a larger neck girth (B), and larger chest girth (C) than wild-type or heterozygous individuals. Males and females are shown separately. +/+, wild-type individuals; *mh/+*, individuals heterozygous for the cys → stop mutation; and *mh/mh,* individuals homozygous for the cys → stop mutation. The center bar indicates the median value and the top and bottom edges of the box delimit the 75th and 25th percentiles, respectively.

The “bully” phenotype displays a simple autosomal recessive mode of inheritance, as all “bullies” resulted from the mating of carriers. The parents have a phenotype of intermediate musculature (Figure 1B). In order to quantify the allelic substitution and dominance effects of the deletion mutation we considered three measures of musculature: mass-to-height ratio, neck girth, and chest girth. For all three measures, heterozygous females (*mh/+)* were intermediate in musculature, *mh/mh* females had the highest measures, and female *+/+* whippets had the lowest measures. Male *mh/+* whippets were more muscular than wild-type males (Figure 3 and Table 1).

**Table 1:**
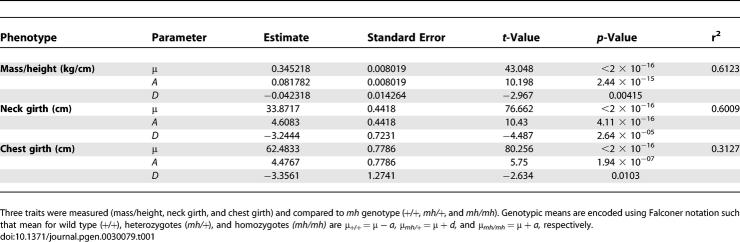
Summary of Least-Squares Regression of Musculature Phenotype on *mh* Genotype

Mass (kg) and height at the withers (cm) information was available for 71 female and 55 male whippets; we analyzed the two sexes separately due to a lack of samples from male *mh/mh* whippets. Two analyses suggest strong statistical support for the idea that the *mh* deletion mutation affects the mass-to-height ratio. First, a nonparametric Kruskal-Wallis one factor Analysis of Variance for females using genotype as a factor (*mh/mh*, *mh/+*, and +/+) was highly significant (*p* ≈ 7.43 × 10^−6^) [10]. Likewise, a two-sample Wilcoxon rank sum test comparing mass-to-height ratio of *mh/*+ and +/+ males was also significant (*p* ≈ 0.00017), with whippets heterozygous for the mutation exhibiting, on average, 17% more mass per cm of height (i.e., 0.333 kg/cm for *mh/*+ as compared to 0.284 kg/cm for +/+ genotypes). Second, we used standard least-squares regression to estimate the allelic substitution (*a* = 0.0817; *p* < 2.44 × 10^−15^) and dominance effects (*d* = −0.0423; *p* = 0.00415) in female whippets and found both parameters were significantly different than zero. These results, along with box plots of the phenotype by genotype class (Figure 3), suggest that the mutation is partially recessive with heterozygotes (*mh/*+) having musculature closer to, but significantly different from, that of wild types (+/+). As reported in Table 1, we found that the *mh* mutation also affected neck and chest girth in a similar fashion in females with a highly significant effect of *MSTN* genotype on phenotype. Overall, we estimate that *mh* explains approximately 60% of the variation in both mass-to-height ratio and neck girth (i.e., *r* ^2^ = 60%) and 31% of the variation in chest girth (Table 1). In male whippets, we also observed a highly significant difference in neck girth (*p* ≈ 0.0013, Wilcoxon rank sum test) and nearly significant difference in chest girth (*p* ≈ 0.11, Wilcoxon rank sum test) among wild type (+/+) and heterozygotes (*mh/*+).

### Association of Heterozygosity with Racing Speed

**Figure 4:**
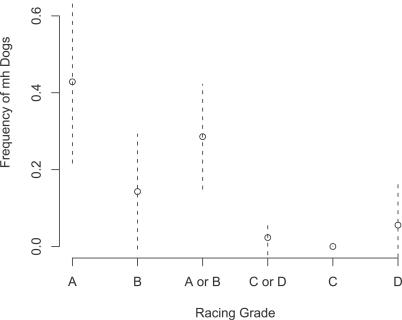
Frequency of Whippets Carrying the Mutation among the Dogs Sampled in Each Racing Grade Dotted lines above and below each point represent 95% confidence intervals on the frequency (± 1.96 standard error on the mean).

We hypothesized that the increased muscle mass of the heterozygotes would allow for increased speed when compared to wild type (+/+) whippets. Analysis of 85 genotyped dogs for which we obtained racing grades revealed an association between a dog's genotype and racing grade using two separate (but not independent) approaches (Figure 4). Only one *mh/mh* dog competed in racing events and it was a grade-A racer, so we included this dog with the heterozygotes and considered the absence or presence of the deletion for all analyses (i.e., we sum across the *mh/+* and *mh/mh* columns in Table 2. First, we find a significant positive correlation between racing grade (A, B, C, and D in order from fastest to slowest) and the frequency of dogs carrying the *mh* deletion in either homozygous or heterozygous state (*p* ≈ 0.00028, τ = 0.3619, Kendall's nonparametric measure). Secondly, standard contingency table analysis reveals strong evidence for heterogeneity in the frequency of dogs carrying the *mh* deletion among racing grade classes (Table 2; *p* = 0.00029, Fisher's exact test with degrees of freedom = 3). Since the 4 × 2 contingency table (i.e., combining columns two and three of Table 2) has three degrees of freedom, it is possible to partition the analysis into three tests of one degree of freedom each in order to identify outlier grades. There are several methods by which this can be accomplished, although they are not independent of each other. For example, 12 of 41 dogs in the fastest two racing grades, A and B, carried the deletion, while just one dog of 43 from the slowest two racing grades, C and D, was a heterozygote (*p* = 0.00073, Fisher's exact test), indicating a strong difference in frequency between (A, B) and (C, D). There is suggestive evidence for a difference between race grades A and B (*p* = 0.086, Fisher's exact test) but no evidence for a difference between C and D (*p* = 0.42, Fisher's exact test). A different approach for dividing the degrees of freedom is to compare A versus (B, C, D) (*p* = 0.00027, Fisher's exact test), C versus (B, D) (*p* = 0.099, Fisher's exact test), and C versus D (*p* = 0.42, Fisher's exact test). Thus, each of these two methods for partitioning the test suggests that the presence of the *mh* mutation strongly influences racing ability.

**Table 2:**
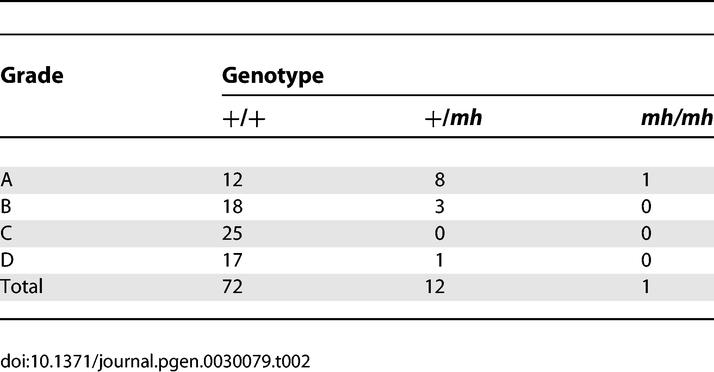
Contingency Table for Racing Grade versus Presence or Absence of the 2-bp Deletion

There was also a marginally significant difference in the mutation genotype frequency in whippets that participated in racing versus conformation events such as breed club regional specialties, where dogs are judged based on their conformation to the physical breed standard (height at the withers or shoulder, head shape, coat color, etc.). Twenty of 119 confirmed racing whippets were heterozygous for the deletion while just two of 42 whippets that competed in conformation events were heterozygous (*p* = 0.038, one-tailed Fisher's exact test).

### Population Structure in the Whippet

To investigate how population substructure within whippets affects our candidate gene analysis, we genotyped 32 unlinked microsatellite markers [11] in 84 whippets with racing grades. Analysis of F_ST_ revealed that whippets exhibit a low, but measurable, degree of population differentiation with higher levels of interbreeding within racing grades. As a result, genetic distance correlates with racing grade so that there is a moderate differentiation between A and B grades (F_ST_ = 0.021), little differentiation between B and C (F_ST_ = 0.0044) or C and D (F_ST_ = 0.0067) racers, and the largest difference between the population of A and D racing dogs (F_ST_ = 0.041). These levels of population differentiation are quite typical for mammalian species and are not surprising, given that whippets are bred to race and positive assortative mating is expected (i.e., fast dogs are bred to fast dogs).

Analysis of the data using Structure [12] and InStruct [13] gave comparable results under a range of cluster numbers from K = 1 to K = 15. Neither program found a clustering that clearly correlated with racing grade. The neighbor-joining tree of the 84 dogs used in this analysis based on genetic similarity (i.e., kinship coefficient) tends to differentiate dogs within the A racing grade from those in other grades, but not in a fully exclusive manner (Figure S1). It also demonstrates that dogs carrying the 2-bp deletion are found on all branches of the tree and are not one another's closest relatives, although they do tend to cluster near one another on the tree.

Logistic regression analysis of 73 alleles at moderate frequencies in the sample confirmed that population substructure may be a potentially confounding effect for association mapping within breeds of dog. In particular, we found that 26 of the alleles (36%) across the 32 loci show a nominal *p*-value of 5% or lower, a huge inflation above the expected 5% (Figure S2A). As a result, many of the 32 loci showed association with racing grade using standard contingency table analysis. However, the association of the 2-bp deletion with racing grade is more significant than all but one of these, suggesting an empirical *p*-value of 1/72 = 0.014 (Figure S2B).

### MSTN Genotypes in Other Breeds and Haplotype Construction

We sequenced exon three of *MSTN* in a set of approximately four dogs each from heavily muscled breeds including the bullmastiff, rottweiler, bulldog, Presa Canario, miniature bull terrier, American Staffordshire terrier, and Staffordshire bull terrier. In addition, we sequenced exon three of *MSTN* in a small set of dogs from breeds known to compete in racing: the greyhound, lurcher, and two mixed-breed dogs (whippet crosses). None of these dogs possessed the 2-bp deletion seen in the whippet.

**Figure 5:**
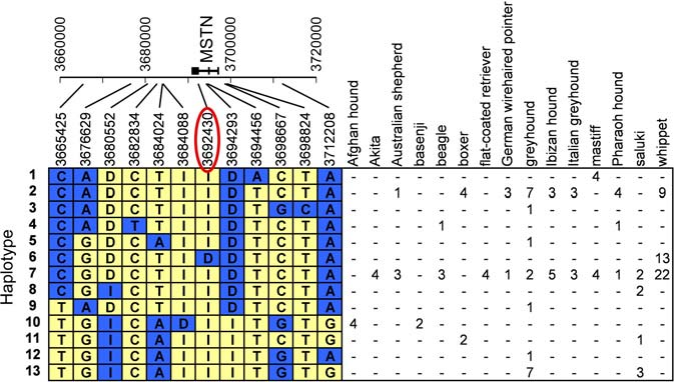
Haplotypes Spanning the *MSTN* Gene Interval from 15 Dog Breeds SNPs and indels were genotyped from sequence reads aligned by Phrap. Insertions and deletions are indicated by I and D, respectively. Haplotypes are shown on the left side with alleles colored yellow if they match the golden jackal, or blue otherwise. The golden jackal is missing data for markers at positions 3,698,667 and 3,698,824 so the most common allele was assumed to be the ancestral allele and was colored yellow. Marker positions and the *MSTN* gene are shown to scale along Chromosome 37 in the plot at the top left. The table to the right shows the number of chromosomes inferred to carry the haplotype in any given breed. Haplotypes were inferred by PHASE independently for each breed and the most likely haplotype pair was selected for each individual. The *mh* mutation (nucleotide position 3,692,430 circled in red) occurs on just one haplotype and is observed only in the whippet breed.

To obtain haplotype information, we sequenced the *MSTN* gene and surrouonding area (Figure 5) in one to ten dogs each from the greyhound, Ibizan hound, Pharaoh hound, Afghan hound, saluki, Italian greyhound, mastiff, boxer, Akita, Basenji, Australian shepherd, beagle, German wirehaired pointer, and flat-coated retriever breeds. A golden jackal was also sequenced to determine the ancestral allele for each dog SNP and insertion/deletion (indel). None of the dogs sequenced from any of the above breeds nor the golden jackal possessed the 2-bp deletion. We discovered 28 SNPs and three indels. For the three indels and eight SNPs that were discovered within 50 kb of the *MSTN* mutation, we inferred haplotypes independently for each breed using PHASE. A total of 13 haplotypes were identified, but only six were observed in more than one breed. Two of these haplotypes are shared across several breeds. The *MSTN* mutation occurs on just one haplotype that is observed only in the whippet (Figure 5). A haplotype observed in 12 breeds differs from this one only at the mutation itself.

## Discussion

The whippet breed was developed in the late 1800s specifically for the sport of racing [3]. Despite its comparatively small stature it is a very fast dog capable of running up to 35 miles per hour [3]. We have discovered a 2-bp deletion in the whippet *MSTN* gene that in the homozygote state results in a double-muscling phenotype commonly referred to as the “bully” whippet. This deletion causes a premature truncation of the protein at amino acid 313, removing the latter 17% of the protein.

*MSTN* has been mapped to canine Chromosome 37 (CFA37) and consists of three exons spanning 5083 bp (http://genome.ucsc.edu). It is highly conserved across species [9] and in the human genome is located on Chromosome two. The gene is a member of the transforming growth factor β family and encodes the myostatin protein. Studies of *Mstn* knockout mice demonstrate that the gene is a negative regulator of skeletal muscle mass [9]. This is the result of a cascading pathway triggered by *MSTN* signaling that prevents myoblast cell progression from the G_1_ to S phase of the cell cycle. *MSTN* therefore controls the total number of muscle fibers by regulating overall myoblast proliferation [14]. In the absence of functional protein, greater numbers of muscle fibers are made [14].

Double muscling has been described in several breeds of cattle [5,6,15–17] and muscular hypertrophy (an increase in muscle-fiber size) has been described in sheep [7]. Muscular hypertrophy has also been described in the domestic cat [18]; however, a deficiency in dystrophin is the cause in this species rather than a mutation in *MSTN*. To date, six different mutations in the bovine *MSTN* gene have been reported to cause double muscling [5,6,15–17]. While all these mutations result in a loss of *MSTN* function, a subset of them and the one we describe here in the whippet likely change the three-dimensional shape of the protein by disrupting the “cysteine knot,” a structure important in the folding of all transforming growth factor β family member proteins [5,9]. The mutation in the whippet also removes nearly 20% of the protein.

We sequenced genomic DNA not only from whippets but also from multiple dogs from each of 14 additional breeds in order to determine the haplotype background on which this mutation arose (Figure 5). In each dog, 15 PCR amplicons that spanned the *MSTN* gene and amplicons spanning known dog SNPs within 50 kb of the *MSTN* mutation were sequenced. Using the resulting data we observed two haplotypes, termed two and seven, that occurred in a large number of breeds and that were identical except at position 3,676,629, which is located outside the gene, 15,801 bp downstream from the 2-bp deletion. The *mh* mutation occurs only on haplotype six, which is identical to haplotype seven except for the deletion itself. Not surprisingly, the golden jackal sequence has only the wild-type allele at the position of the mutation, indicating the *mh* allele represents the derived state. We conclude therefore that haplotype six likely derives from haplotype seven (Figure 5). Haplotype seven is the most common and widely dispersed haplotype spanning the gene and was found in 12 out of 15 breeds sequenced. Interestingly, we did not observe haplotype seven in the Afghan hound, Basenji, or boxer.

Our data do not exclude the possibility that the mutation occurs in breeds other than the whippet. However, we screened for the 2-bp deletion in several mastiff type breeds (rottweiler, bulldog, Presa Canario, miniature bull terrier, American Staffordshire terrier, Staffordshire bull terrier, and bullmastiff) and did not find it. These data argue that the changes in musculature exhibited by the whippet are unique and caused by the effects on *MSTN* associated with the deletion described in this study.

An excess of the *mh/+* genotype was observed among the fastest racers, as defined by the highest racing grade achieved during a dog's career. This demonstrates that the heterozygote state carries a performance-enhancing polymorphism that provides a competitive edge. The optimal study of racing performance would use the racing points acquired by each whippet during their career as a quantitative measure of performance. However a dog's total career points are a function of the number of races run throughout their career and, as such, whippets of different ages are not easily compared. To compensate, the total number of points accrued over a lifetime of racing could be averaged over the number of races entered. However, as dogs age their performance declines. Some owners stop racing their dogs after their performance declines while others continue to race their dogs for months or even years longer. Using the average number of points accrued during a specific year of the dog's life, for instance age two or three, presents similar problems. Dogs reach their racing prime at different ages and the number of points will always reflect the number of races entered. While an average is satisfactory if many races are run in a given year, the average will be inaccurate if few races are run.

While cattle breeders have long selected for individuals that are homozygous for mutations in *MSTN* because of their increased musculature, which is optimal for beef production, this is the first example of breeders unknowingly selecting for individuals with a single polymorphism that increases athletic performance. Of interest, the trait appears to confer an undesirable appearance upon dogs competing in conformation. Only two *mh/*+ dogs were found among the dogs reported to compete in conformation events, and those dogs were reported to show poorly. This is consistent with the association seen between a dog's genotype and their relative muscle mass as defined by either a ratio of mass (kg) to height at the withers (cm) (*p* = 7.43 × 10^−6^; Kruskal-Wallis test) (Figure 3A) or the direct measure of an individual's neck girth (*p* = 3.47 × 10^−5^; Kruskal-Wallis test) (Figure 3B) or chest girth (*p =* 0.001462; Kruskal-Wallis test) (Figure 3C). We acknowledge that there are more accurate methods to measure muscle mass. However, many of these methods are either invasive, such as a muscle biopsy, or would need to be conducted post-mortem, neither of which was an option. These measurements were not designed to specifically eliminate contributions from body fat. However, obesity is rare in the whippet; indeed, the breed is characterized by an overall low body fat content. Thus, these measurements are the best achievable metrics of the phenotype.

Greyhounds and whippets share a common ancestral gene pool and as a result the breeds are difficult to separate in genetic clustering analyses [11]. This, together with the fact that both were bred to excel at racing, suggested that the mutation might also be found in racing greyhounds. However, none of the greyhounds tested carried the mutation. There are three possible explanations for this result. First, an insufficient number of samples have been tested if the mutant allele is relatively rare in the greyhound population. Second, the mutation may only be present in a subset of greyhound lines, none of which were among those tested. Finally, the mutation may not be carried in the greyhound population at all, indicating that it is a relatively new mutation in the purebred dog population. This may be because the mutation offers no advantage to greyhound racers. Indeed, it may even be disadvantageous. Studies of muscle composition in *Mstn* knockout mice demonstrate a higher proportion of both fast type II and glycolytic fibers, versus slow type I and oxidative fibers when compared to wild-type mice [19]. While this change in muscle composition may offer an advantage to whippets, which typically race a short sprint of 200–300 m, it may be disadvantageous to greyhounds, whose races extend to 900 m and where endurance is more important. In addition, Belgian Blue cattle that are homozygous for a *MSTN* mutation display a decrease in the size of several organs, including the lungs [20]. If heterozygous dogs have even a slightly reduced lung capacity, it is possible that a *MSTN* mutation would actually be disadvantageous for racing longer distances as greyhounds do. Finally, it remains to be determined whether additional health problems are associated with being a carrier of this mutation.

We examined the microsatellite data set for evidence of population substructure and found that there is not random gene flow across the racing classes. All groups display positive F_ST_ values with the greatest found between the grade A racers and all others. This is not unexpected. The very presence of the “bully” phenotype is evidence that breeders choose to mate dogs with increased musculature to one another. Reducing the mating population of a breed to a small proportion of the whole population has consequences, particularly for genetic mapping of complex traits. This is evidenced by our analysis of the same marker set for association with racing grade. While we find low *p*-values at many of the alleles, only one of 73 had a *p*-value smaller than the 2-bp deletion, confirming that the association between racing grade and the *MSTN* mutation is not simply a spurious result of population structure. Overall, these results suggest that the population structure within breeds is likely to have an important confounding effect on association mapping in the domestic dog.

Our findings have implications for competitive and professional sports. Here, we show that a disruption in the function of the *MSTN* gene increases an individual's overall athletic performance in a robust and measurable way. To date, the muscular hypertrophy phenotype has been described in a single human child [8]. This child possessed two copies of a G-to-A transition in the noncoding region of the human *MSTN* gene. This mutation results in the mis-splicing of precursor mRNA, which most likely truncates the myostatin protein. The child's mother, a former professional athlete, was heterozygous for this mutation and also appeared muscular, although not to the same degree as her child. Perhaps additional mutations in *MSTN* have yet to be discovered in other species that competitively race, such as the horse or humans. As discussed by others [21,22], human athletes could undergo so-called gene doping via disruption of *MSTN*. The potential to increase an athlete's performance by disrupting *MSTN* either by natural or perhaps artificial means could change the face of competitive human and canine athletics. Given the poorly understood consequences for overall health and well-being, caution should be exercised when acting upon these results.

## Materials and Methods

### Sample collection.

An initial set of 22 whole-blood samples were collected from whippets that participated in racing, conformation, or were simply privately owned pets. Of these 22 dogs, four were reported by owners to be “bullies,” five dogs had either sired or whelped a “bully,” and the owners of the remaining 13 stated there was no known family history of “bullies” in their dog's pedigree. After initial analysis of these 22 samples an additional 46 whole-blood samples and 100 buccal swabs were collected from a mixture of racing, conformation, and pet whippets. No restrictions were placed on age, gender, or relatedness of the dogs sampled. The dog's sex was recorded for 165 (98%) of the dogs (74 males and 91 females). Samples were collected both by mail and at sanctioned Whippet Racing Association (WRA), National Oval Track Racing Association (NOTRA), and Continental Whippet Alliance (CWA) racing events. Blood samples from each dog were collected as whole blood in ACD tubes. Buccal swabs were collected using standard protocols with Cytosoft cytology brushes (Medical Packaging Corporation, http://www.medicalpackaging.com). DNA was extracted from the brushes using a QIAamp Blood Mini kit (Qiagen, http://www.qiagen.com) following the manufacturer's protocol. DNA was extracted from the blood samples using a standard phenol/chloroform extraction method [23].

DNA samples were also collected from 33 greyhounds, two mixed-breed dogs (whippet crosses), seven lurchers, five each of bullmastiffs, rottweilers, and bulldogs, four each of Presa Canarios, miniature bull terriers, mastiffs, Staffordshire bull terriers, Ibizan hounds, and salukis, three each of American Staffordshire terriers, Italian greyhounds, boxers, and Pharaoh hounds, two each of Akitas, Afghan hounds, Australian shepherds, beagles, flat-coated retrievers, and German wirehaired pointers, a single Basenji, and a single golden jackal. Samples were either received as DNA from collaborators or DNA was extracted by the aforementioned methods after collection at dog shows, events, or provided directly by owners and breeders. Informed consent was obtained for all newly collected samples and all protocols were approved by the Animal Care and Use Committee of the Intramural Program of the National Human Genome Research Institute at the National Institutes of Health.

### Phenotype assessment.

Owners of whippets were asked to provide detailed information about their dogs including American Kennel Club or other registration number, pedigree information, and the events in which their dogs participated. Front and side photos of individual dogs were obtained for comparison. The dog's mass and height at the withers were recorded for 126 dogs (55 males and 71 females). A set of body measurements including neck girth and chest girth were also collected from 137 of the whippets (61 male and 76 female) either by owners or laboratory members as described (N. B. Sutter, D. S. Mosher, and E. A. Ostrander; personal communication).

There are four organizations that govern whippet racing in the United States; straight racing is sponsored by the WRA, CWA, and the North American Whippet Racing Association (NAWRA), while oval racing is sponsored by NOTRA. The standard track length of a straight race is 182.88 m, while the standard oval track is 350 m. A race meet is composed of four heats. Based on their placement in all four heats, dogs are given a total meet score. A dog's racing grade is a simplified assessment of its performance over the last three meets. As a result, a dog's racing grade will vary throughout its career as grade is reassessed following each meet. We used the WRA racing grades: Grade A is 15.0–29.0 points, grade B is 10.0–14.9 points, grade C is 4.0–9.9 points, and grade D is 0–3.9 points (http://www.whippetracing.org/Rules/2006/2006Chapter5.htm). We categorized dogs based on the highest grade ever achieved. Grades for 85 racing whippets were obtained from the WRA website (http://www.whippetracing.org).

### Regression analysis.

We employed least-squares regression to estimate the allelic substitution *(a)* and dominance *(d)* effects of the mutation using a Falconer parameterization for genotypic means [24]. Briefly, we assumed the phenotypes within genotypic classes are normally distributed with mean for wild type (+/+), heterozygotes (*mh/*+), and homozygotes *(mh/mh)* of μ_+/+_ = μ − *a,* μ*_mh/_*_+_ = μ + *d,* and μ*_mh/mh_* = μ + *a*, and with common variance σ^2^ within classes. This amounts to using two “dummy” variables to encode the design matrix for the regression with values (−1, 0, and 1) and (0, 1, and 0) for the genotype classes (+/+, +/*mh,* and *mh/mh*). All measurements were normalized (i.e., mean subtracted and observations divided by the standard deviation) and Q-Q plots inspected visually to assess the appropriateness of normality assumption within genotypic classes. All statistical analyses were carried out using R 2.4.1 (www.r-project.org).

### Analysis of population structure.

We genotyped 32 unlinked microsatellite markers [11] in 84 whippets with racing grades. All loci were found to be variable with a total of 135 alleles segregating across all markers. We used *GDA* [25] to calculate Wright's fixation index (F_ST_) and estimated kinship coefficients using Ritland's method [26] and Rousset's genetic distance [27] using Spagedi 1.2f [28]. Population clustering was assayed using the Bayesian clustering algorithm Structure [12], which assumes Hardy-Weinberg equilibrium within clusters and InStruct [13], a Structure-like algorithm that estimates a generalized inbreeding coefficient for each cluster. A neighbor-joining tree was constructed by using Rousset's genetic distance [27] as input into Phylip 3.66 (http://evolution.genetics.washington.edu/phylip.html) and racing grade state was traced across the tree using MacClade 4.0 (http://macclade.org/) (Figure S1).

We analyzed the control loci for association between genotype and racing grade using standard logistic regression analysis for differentiating A racers from B, C, or D racers. For each control locus, we fit a saturated model with *n_l_* parameters, where *n_l_* is the number of microsatellite alleles found at locus *l* (each analysis had (*n_l_* − 1) allele effects and one intercept parameter). Figure S2 shows the distribution of *p*-values for the 73 allele effects with *p*-values below 0.98. In total there were (135 − 32) = 103 independently estimated allele effects, but for 30 of these there was little power to detect an effect because so few dogs carried the allele.

### Sequencing of the MSTN gene and SNP genotyping.

The entire canine *MSTN* gene was sequenced except for a 1,039-bp GC-rich region in intron one. Twelve pairs of overlapping primers covering the remaining regions of the gene and three primer pairs for SNP genotyping were designed using Primer 3 software [29] (http://frodo.wi.mit.edu/cgi-bin/primer3/primer3_www.cgi). Exon/intron boun-daries were based on the complete canine *MSTN* mRNA sequence. The resulting amplicons averaged 700 bp in length (653 to 799 bp). PCR was performed in a total volume of 10 μl containing 10 ng of dog DNA, 1× reaction buffer (Applied Biosystems, http://www.appliedbiosystems.com), 0.1 mM dNTP (Promega, http://www.promega.com), 1.5 mM MgCl_2_, 0.5 U of AmpliTaq Gold polymerase (Applied Biosystems) and 0.03 μM each specific primer. Touchdown PCR was carried out as follows: 7 min at 95 ^°^C followed by 20 cycles of 30 s at 95 ^°^C, 30 s at annealing temperature (beginning at 61 ^°^C and decreasing 0.5 ^°^C per cycle) and 30 s at 72 ^°^C then 20 cycles of 30 s at 95 ^°^C, 30 s at 51 ^°^C and 30 s at 72 ^°^C and a final extension phase for 3 min at 72^ °^C.

The resulting PCR products were sequenced using Big Dye version 3.1 on an ABI 3730xl capillary sequencer (Applied Biosystems). Sequence reads were aligned and analyzed using Phred, Phrap, and Consed [30,31,32]. Polyphred [33] was used to assist in the identification of all SNPs and indel polymorphisms.

## Supporting Information

Figure S1 Neighbor-Joining Tree for 84 Whippets with Racing Grade The tree was estimated using Rousset's genetic distance [27] from 32 unlinked microsatellite loci with racing grade traced using MacClade 4.0 (red = A, orange = B, aqua = C, and purple = D). Unique identifiers and racing grade are listed above each dog. Black boxes denote dogs that carry the 2-bp deletion and white boxes denote +/+ dogs.
(32 KB PDF)

Figure S2 Logistic Regression of Racing Grade on Genotype The distribution of nominal *p*-values for logistic regression of racing grade (A versus B, C, or D) on genotype is shown for 73 alleles spanning 32 unlinked microsatellite loci.
(A) Empirical cumulative distribution of *p*-values (open circles). The solid line represents the expected distribution under the null hypothesis of no association between genotype and racing grade.
(B) Histogram of *p*-values on a log scale showing the location of the *p*-value for the 2-bp deletion.
(132 KB PDF)

### Accession Numbers

The Genbank (http://www.ncbi.nlm.nih.gov/gquery/gquery.fcgi) accession number for the complete canine *MSTN* mRNA sequence is AY367768. The Genbank accession number for the *MSTN* protein is NP_01002959.

## Abbreviations

indel: insertion/deletion
*MSTN,*: myostatin
