# Supplementary figures and images for: A Mutation in the Myostatin Gene Increases Muscle Mass and Enhances Racing Performance in Heterozygote Dogs
Source: PLoS Genet. 2007 May 25;3(5):e79. doi: 10.1371/journal.pgen.0030079 (PMC1877876; doi:10.1371/journal.pgen.0030079)

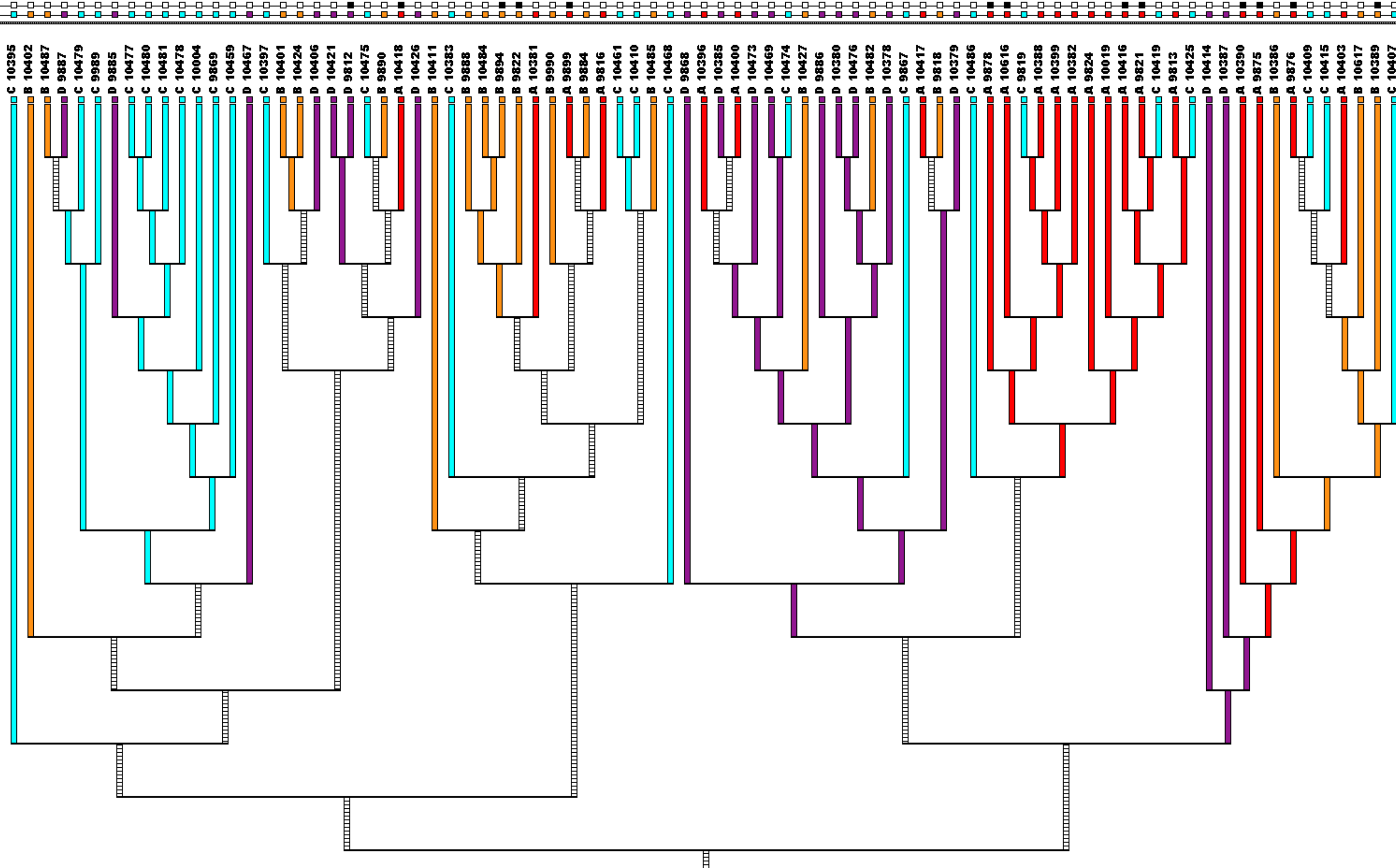

Supplement: Figure S1 — The tree was estimated using Rousset's genetic distance [27] from 32 unlinked microsatellite loci with racing grade traced using MacClade 4.0 (red = A, orange = B, aqua = C, and purple = D). Unique identifiers and racing grade are listed above each dog. Black boxes denote dogs that carry the 2-bp deletion and white boxes denote +/+ dogs. (32 KB PDF) [file pgen.0030079.sg001.pdf]

A.

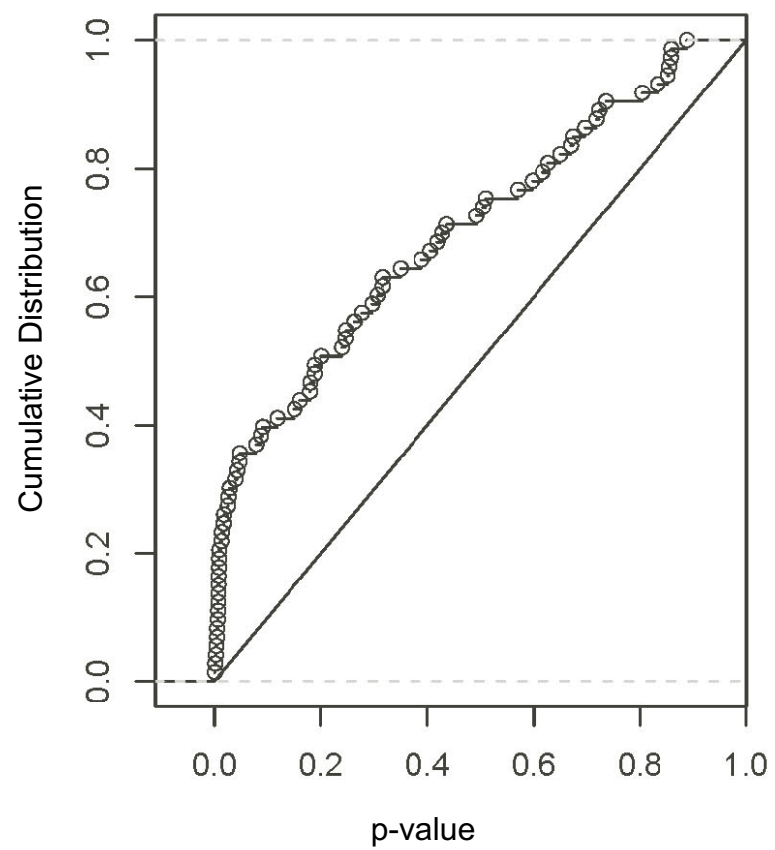

B.

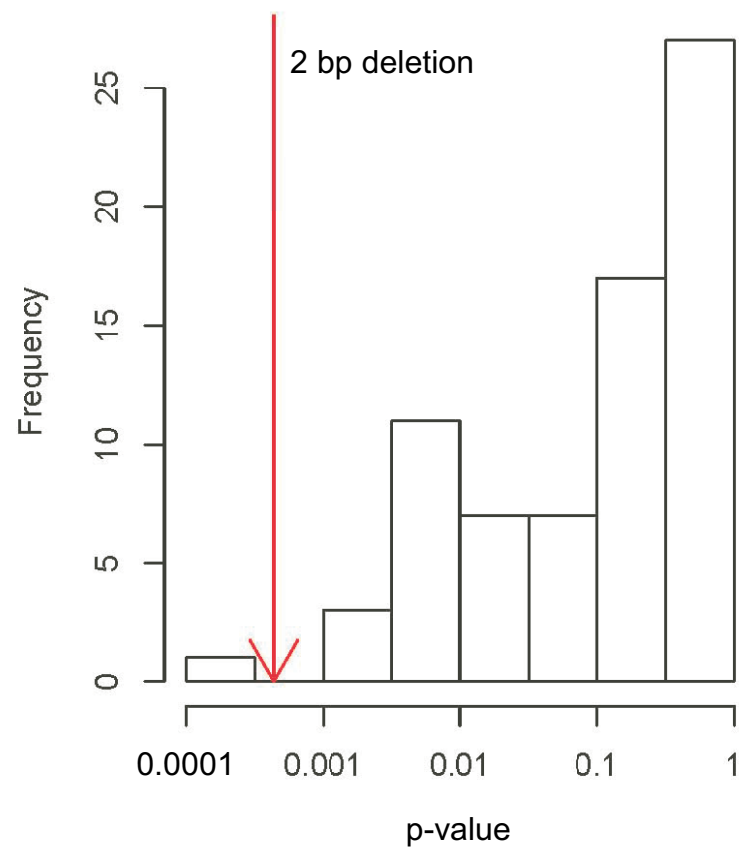

Supplement: Figure S2 — The distribution of nominal p-values for logistic regression of racing grade (A versus B, C, or D) on genotype is shown for 73 alleles spanning 32 unlinked microsatellite loci. (A) Empirical cumulative distribution of p-values (open circles). The solid line represents the expected distribution under the null hypothesis of no association between genotype and racing grade. (B) Histogram of p-values on a log scale showing the location of the p-value for the 2-bp deletion. (132 KB PDF) [file pgen.0030079.sg002.pdf]
